# *VDR* Gene Polymorphisms (*BsmI*, *FokI*, *TaqI*, *ApaI*) in Total Hip Arthroplasty Outcome Patients

**DOI:** 10.3390/ijms25158225

**Published:** 2024-07-27

**Authors:** Dominika Rozmus, Ewa Fiedorowicz, Roman Grzybowski, Janusz Płomiński, Anna Cieślińska

**Affiliations:** 1 Department of Biochemistry, Faculty of Biology and Biotechnology, University of Warmia and Mazury in Olsztyn, 1A Oczapowskiego Street, 10-719 Olsztyn, Poland; dominika.rozmus@uwm.edu.pl (D.R.); ewa.kuzbida@uwm.edu.pl (E.F.); 2 Department of Orthopedics and Traumatology, Collegium Medicum, University of Warmia and Mazury in Olsztyn, Aleja Warszawska 30, 11-041 Olsztyn, Poland; roman.grzybowski@uwm.edu.pl; 3 Department of Orthopedics, Centre of Postgraduate Medical Education, Gruca Orthopaedic and Trauma Teaching Hospital, Konarskiego 13, 05-400 Otwock, Poland; plominsky@poczta.onet.pl

**Keywords:** VDR, vitamin D receptor, vitamin D, loosening of hip prothesis, arthroscopy

## Abstract

A total hip arthroplasty (THA) can improve quality of life, but loosening of the hip prosthesis is a complex problem in which vitamin D may also play a role. The Vitamin D Receptor (VDR) is involved in the response of cells to the action of vitamin D, and its genetic variability raises the question of whether individual differences could influence the risk of prosthesis loosening. The aim of this study was to investigate the relationship between *VDR* single nucleotide polymorphisms (SNPs) (*ApaI*, *BsmI*, *FokI* and *TaqI*) and the serum VDR and 25(OH)D levels in three groups of patients: (1) arthroscopy patients after THA without loosening of the prosthesis (CA—Control Arthroplasty), (2) patients after THA with loosened hip prostheses (L—Loosening) and (3) the control group (C—Control). Our results suggest that the genotypes *tt* of *TaqI*, *BB* of *BsmI*, and *FF* of *FokI* may influence the *VDR* effect in patients with loosened protheses. Our results showed that the ACAC haplotype (AtBF) was over two times more frequent in the L group than in CA + C: OR =2.35 [95% CI 1.44–3.83; *p* = 0.001]. There was no significant correlation between the VDR and serum 25(OH)D levels, but there were differences between studied groups.

## 1. Introduction

Total hip arthroplasty (THA) is a procedure that has been performed for more than 100 years and has remained one of the most successful orthopedic operations since its inception [1]. The increasing incidence of osteoporosis and bone-related trauma in the elderly population has led to an increase in hip fractures and the risk of patient mortality [2]. One of the options in cases of injury is total hip arthroplasty (THA), in which the femoral head and acetabulum are replaced with prosthetic components [3]. This medical procedure improves quality of life but is not without complications. THA complications/adverse events and their definitions have been endorsed by the Hip Society, including bleeding, wound complication, thromboembolic disease, neural deficit, vascular injury, dislocation/instability, periprosthetic fracture, abductor muscle avulsion, deep periprosthetic joint infection, heterotopic ossification, wear of bearing surfaces, osteolysis, implant loosening, cup dislodgement, implant fracture, reoperation, revision, readmission and death [4]. According to statistics, the main causes of prosthesis loosening are inflammatory reactions (septic in 7.5%/aseptic in 55.2%), dislocations and prosthesis fractures (18.8%) and other surgical errors (3.8%) [5,6]. Another study examined two groups of patients, (1) THA and (2) THA revision (rTHA), with the most common concomitant diseases in both groups being hypertension and chronic lung disease. The rTHA indications included dislocation/instability (21.85%), followed by mechanical loosening (19.74%), other mechanical complications (17.38%), and infection (15.10%). The five most common concomitant diseases in the THA group are hypertension (60.46%), obesity (15.11%), chronic lung disease (14.37%), hypothyroidism (13.68%) and uncomplicated diabetes (13.67%). In rTHA, the five most prevalent comorbidities are hypertension (59.74%), deficiency anemia (17.51%), chronic lung disease (16.92%), fluid/electrolyte disorders (15.74%) and hypothyroidism (14.81%) [7]. The problem of inflammation and the strong immune reaction caused by wearing the prostheses has not yet been solved, and research to date has focused on the local processes around the prosthetic components. Recent reports suggest that the quality and metabolism of bone tissue influence the process of osseointegration [8,9], as does a disturbance in calcium phosphate metabolism related to the role of vitamin D in the human body [10,11]. Osseointegration was originally defined as a direct structural and functional connection between bone and the surface of an implant. Osseointegration can be described as the final step in a cascade of processes involved in bone healing around implants. Osseointegration and implant success are influenced by systemic factors such as metabolic bone diseases [12,13].

**Figure 1:**
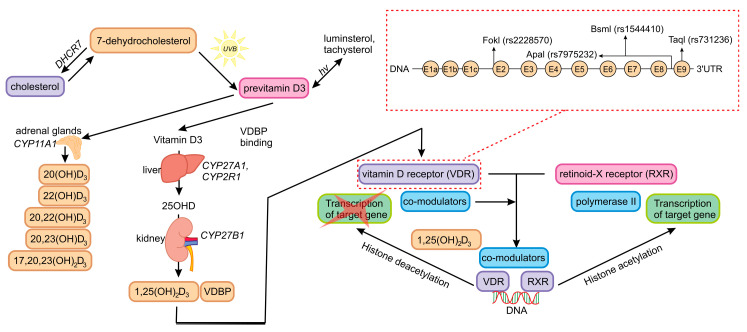
Metabolic pathway of vitamin D and the role of VDR (Vitamin D Receptor) in signaling based on Płomiński et al. (2022), Slominski A.T. (2024), Holick (2024) and Slominski R.M. (2021) [16,19,20,21].

The role of vitamin D, an important element of the endocrine system, in bone health is well documented. It controls calcium and phosphate homeostasis throughout the body, as well as, together with parathyroid hormone, bone mineralization [14,15,16,17]. As a secosteroid prohormone with wide-ranging regulatory effects, it influences gene expression and affects cell proliferation, differentiation and activation [18]. The metabolic pathway of vitamin D activation is shown in Figure 1 below.

In orthopedic surgery, vitamin D levels correlate with bone density, reduction in the rate of osteoporotic fractures, integration of prostheses and improved neuromuscular function [22]. Vitamin D deficiency is associated with numerous diseases ranging from bone mineralization disorders to chronic diseases such as diabetes, cardiovascular disease, cancer, multiple sclerosis, rheumatoid arthritis and tuberculosis [23,24,25,26].

Epidemiological data have shown that an increasing number of orthopedic patients are at risk of vitamin D deficiency, which can have far-reaching consequences in terms of bone healing and fracture risk [22]. Vitamin D modulates immune reactions, which are of critical importance in connection with the loosening of protheses. Hypovitaminosis has been found in patients with total hip and also with knee arthroplasty [10]. In addition, patients suffering from periprosthetic joint infection of the hip and knee appear to have lower vitamin D levels than patients with aseptic loosening of the implants [11].

The Vitamin D Receptor (VDR) is a component of the metabolic pathway involved in the cellular response to vitamin D action [17]. As a member of the nuclear receptor superfamily, it functions as a ligand-induced transcription factor [17,27] and binds the active vitamin D metabolite 1,25-dihydroxycholecalciferol (1,25(OH)_2_D) to the target cell [17]. *VDR* is expressed in various tissues, including intestinal epithelial cells, renal tubules, parathyroid cells, keratinocytes of the skin, breast epithelium, the pancreas, the pituitary gland, osteoblasts, chondrocytes, monocytes, macrophages, T lymphocytes and germinal tissue. Its role in the regulation of gene expression involved in organ development, cell proliferation and differentiation, calcium and phosphate homeostasis in bone metabolism, immune system function and the detoxification of xenobiotics is well documented [28,29]. Although Figure 1 presents mostly canonical vitamin D metabolic pathway activation, novel pathways have already been discovered [30]. They include the role of lumisterol and tachysterol, which may be activated by CYP11A1, and their metabolites act not only on VDR but also other nuclear receptors, such as LXRα/β, RAR-related orphan receptor α/γ and peroxisome proliferator–activated receptor-γ [21,30,31].

The *VDR* gene, which consists of 12 exons, is located on the long arm of the 12 chromosome (12q13.11) [32]. It has several polymorphisms, and the most common SNP-like alterations in the *VDR* have been found and named as *FokI* (rs2228570), *ApaI* (rs7975232), *BsmI* (rs1544410) and *TaqI* (rs731236) [33]. A standardized nomenclature of *VDR* polymorphisms is shown in Table 1.

The polymorphisms and the extent of expression of genes involved in the vitamin D metabolic pathway may be an important element in the effect of vitamin D on the human body, including disorders in the skeletal system and the problem of loosening of orthopedic prostheses [34,35,36,37,38].

The genetic variability of the *VDR* gene raises the question of whether individual differences could influence the risk of hip prosthesis loosening. The study is of crucial importance in the context of hip replacements, as vitamin D deficiency is particularly common in older populations. Therefore, this study aims to gain new insights by investigating the relationship between *VDR* polymorphisms (*ApaI*, *BsmI*, *FokI* and *TaqI*) and serum VDR protein levels in three groups of patients: (1) a group of arthroscopy patients after THA without prosthesis loosening, (2) patients after THA with a loosened hip prosthesis and (3) the control group (healthy subjects).

## 2. Results

### 2.1. ORs and Allele/Genotype Frequencies

The observed genotype frequencies of *ApaI*, *BsmI* and *TaqI* polymorphisms in *VDR* in the study groups corresponded to the Hardy–Weinberg equilibrium. Our results suggest that the *tt* of *TaqI*, *BB* of *BsmI* and *FF* of *FokI* genotypes may influence the *VDR* action in loosening prothesis patients. Table 2, Table 3, Table 4 and Table 5 show the detailed individual genotypes, allele frequencies and the associations with prosthesis loosening for each investigated SNP.

### 2.2. Linkage Disequilibrium (LD)

LD refers to the non-random association of alleles at a pair of genetic loci [39].

**Figure 2:**
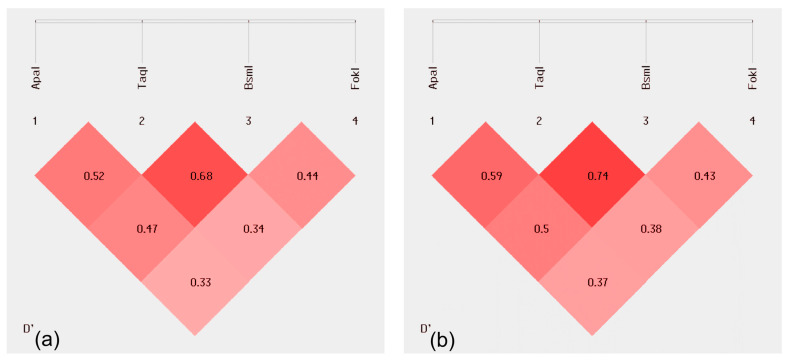
Linkage disequilibrium between *Apa*, *Taq*, *Bsm* and *FokI* in all studied groups: (**a**) L vs. CA + C, (**b**) L vs. CA. The more intense color, the higher LD result is.

The results show that *ApaI* and *TaqI* (LD = 0.59) and *TaqI* and *BsmI* (LD = 0.74) have a higher association frequency than expected in the L vs. C group and *TaqI* with *BsmI* (LD = 0.68) in L vs. CA + C. Figure 2 shows the above results.

### 2.3. Haplotype Analysis of Loci

A haplotype analysis of the loci is presented in Table 6 (L vs. CA + C) and Table 7 (L vs. C).

Haplotype analysis of the loci *ApaI*, *TaqI*, *BsmI* and *FokI* SNPs in the *VDR* gene in groups L vs. C showed that *ACAC* haplotype (*AtBF*) was over two times more frequent in the L group than CA + C: OR = 2.35 [95% CI 1.44–3.83; *p* = 0.001] and than C group OR = 2.50 [1.50~4.15; *p* = 0.0008]. The *CTGT* (*aTbf*) haplotype showed a protective effect against THA (C group), while it was less frequent in the L group (OR = 0.54; 95%CI = 0.32–0.90; *p* = 0.017]. The *ATGT* (*ATbf*) haplotype was less frequent in the L group (OR = 0.42; 95%CI = 0.18–0.98; *p* = 0.039) compared to CA + C and C (OR = 0.38; 95%CI =0.16–0.91; *p* = 0.024).

### 2.4. VDR and 25(OH)D Levels and Correlations

**Figure 3:**
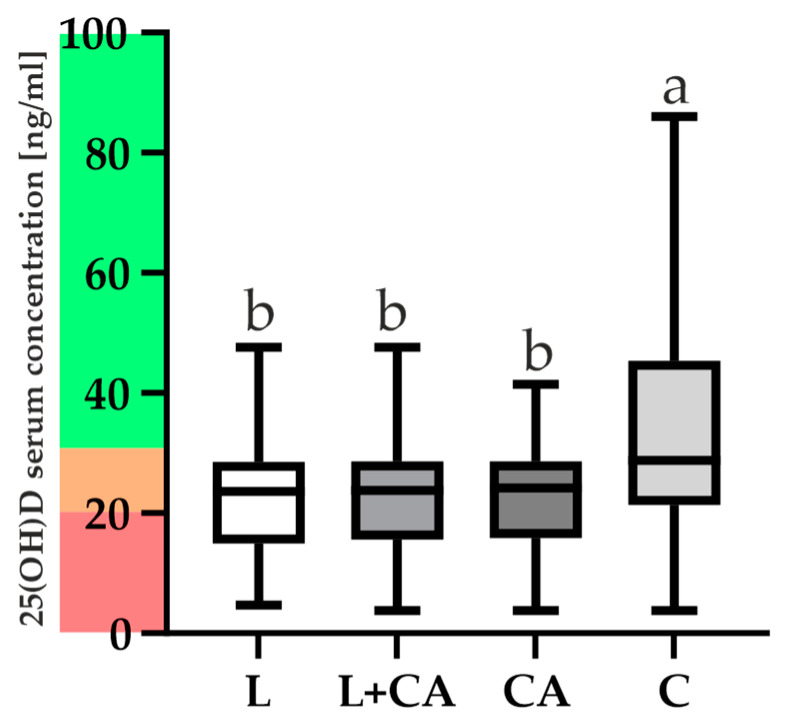
Comparison of 25(OH)D serum concentration in all studied groups with difference analysis, where letter b presents the groups that do not differ from each other and letter a differs from groups marked with letter b. The figure also presents vitamin D concentrations on a scale: red—deficiency, orange—insufficiency, green—sufficiency. Whiskers of the boxplot show the min to max range of concentrations, while a solid line indicates the median.

Our results showed higher serum 25(OH)D levels in the C group (Figure 3) compared to the group of arthroscopy patients after THA without loosening of the prosthesis and patients after THA with loosened hip prostheses. The comparison between the groups was carried out using ANOVA and the non-parametric Tukey (HSD) test. In 25(OH)D, one-way ANOVA showed the following differences between groups: F = 30.05, *p* < 0.0001. The Tukey (HSD) test results showed differences between L vs. C, L + CA vs. C and CA vs. C (*p* < 0.0001). There were no significant differences between other groups (*p* > 0.7112).

There were differences in 25(OH)D concentrations between groups which were marked as a and b (Figure 3).

Our results show that the mean of VDR serum level in the C group is lower compared to other groups. In terms of VDR concentration, one-way ANOVA showed there are differences between groups: F =43.19, *p* < 0.0001, df =3. Tukey (HSD) test results showed there are differences between L vs. C, L vs. CA + C and CA vs. C (*p* < 0.0001). Differences were not shown among other groups (*p* > 0.8524). 

**Figure 4:**
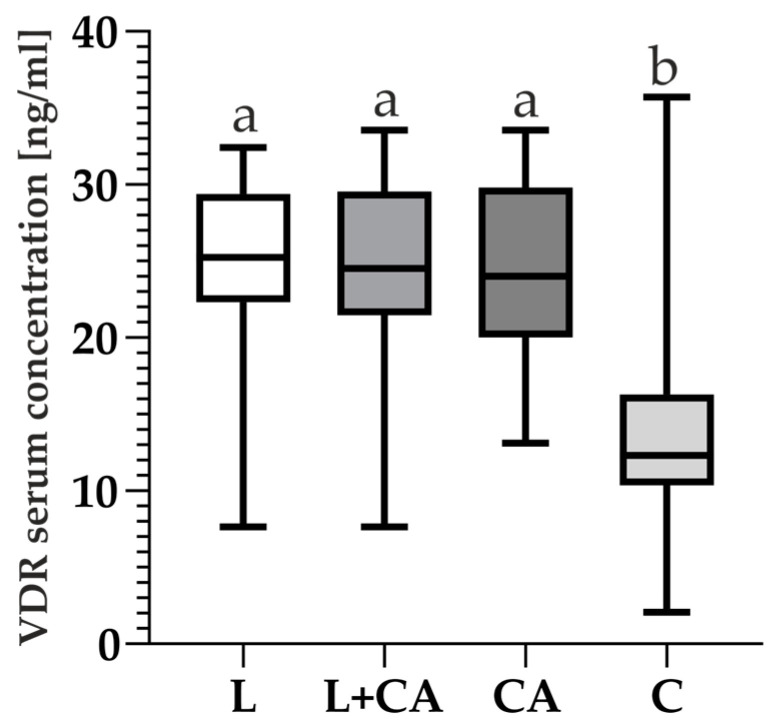
Comparison of VDR (Vitamin D Receptor) serum concentration in all studied groups with difference analysis, where letter a presents groups that do not differ from each other, while letter b differs from groups marked with letter a. Whiskers of the boxplot show the min to max range of concentrations, while a solid line indicates the median.

There were differences in VDR concentrations between groups which were marked as a and b (Figure 4).

**Figure 5:**
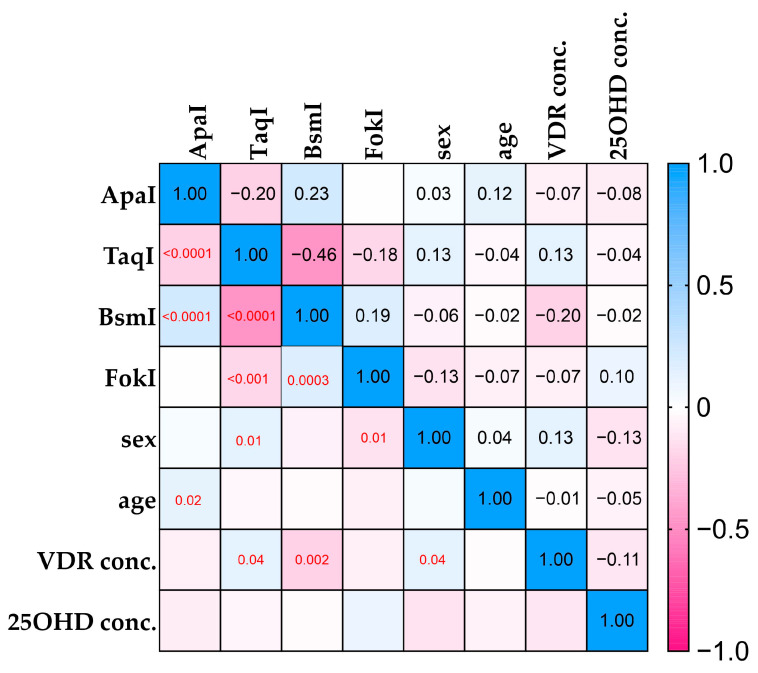
Heatmap of Spearman’s rank correlation with *p*-values (red) marked only for significant results.

The Spearman’s rank correlation presented in Figure 5 shows correlations between polymorphisms and 25(OH)D and VDR serum concentrations. The more positive the correlation, the bluer the graph, and the more negative the correlation, the pinker it is. Significant correlations are also marked with a red *p*-value on the graph. *BsmI* and *TaqI* show a strong correlation, which is also demonstrated by LD. VDR and 25(OH)D show a weak negative correlation but with no significance in *p*-value.

## 3. Discussion

The loosening of prostheses can be caused by many factors. These include a reduction in bone mineral density (BMD) [40], unrecognized osteoporosis (OP) or vitamin D deficiency [41]. Vitamin D is important for bone development, skeletal remodeling and fracture repair, which is why the vitamin D metabolic pathway should be investigated in more detail. Higher vitamin D levels are beneficial, and adequate calcium and vitamin D intake to prevent secondary hyperparathyroidism, leading to further bone loss, is the best indicator of adequate calcium and vitamin D intake in any patient [42]. To our knowledge, there are no studies on polymorphisms in the VDR gene in THA patients. Therefore, we base our results on data from publications related to bone diseases (mainly BMD). Some studies clearly showed that patients with a vitamin D deficiency had worse preoperative Harris hip scores (Mann–Whitney test, *p* = 0.018) and were significantly less likely to have an excellent outcome after total hip arthroplasty (*p* = 0.038). Vitamin D levels were found to be positively associated with both pre- and post-operative Harris hip scores [43].

The study by Berg et al. showed that the *VDR* genotype in a highly endemic osteoporosis area did not predict premenopausal bone mass, postmenopausal bone loss or later osteoporosis and fracture risk [44].

Regarding polymorphisms of the *VDR* gene in a Caucasian population, the meta-analysis in 2020 [45] shows that *FokI* is associated with osteoporosis (OR_dominant_ = 1.15; 95% CI = 0.96–1.38. *p* = 0.12), as is *TaqI* (OR_dominant_ = 1.31; 95% CI = 1.12–1.53. *p* = 0.001). In that study, no other polymorphism was associated with the disease [45].

The study of Vandevyver et al. [46] showed no significant correlation between the *VDR* polymorphisms of *BsmI*, *ApaI*, *TaqI* and BMD, while Nguyen et al. demonstrated a significant correlation between the *TaqI* polymorphism and BMD [47], with the *TaqI*-*CC* (*tt*) genotype being associated with an increased risk of hip fracture (OR = 2.6; 95%CI = 1.2–5.3) and BMD.

In Poland, Horst-Sikorska et al. examined a group of 187 patients with osteoporosis and compared them with 19 healthy subjects. The *VDR* polymorphism *TaqI* with T allele was significantly associated with BMD, while the *ApaI* aa variant, the *BsmI* bb variant and the TT *Taq* variant occurred most frequently in patients with a higher of fracture risk in the Polish osteoporotic population [48].

Another study was conducted in Turkey by Uysal et al. in which postmenopausal women were recruited (*n* = 200 with osteoporosis, *n* = 146 healthy controls). The study confirmed differences in the distribution of *BsmI* frequencies, resembling Caucasians in the Turkish population, while the frequency of the *Taq* genotype did not resemble either Caucasians or Asians. The *VDR* haplotypes *bbAATT* and *bbTtAa* were more frequent in the osteoporosis group compared to the healthy control group [49]. The results of our studies show that the *TT* genotype of *TaqI*, the *BB* of *BsmI* and the *ff* of *FokI* were more frequent in the control group (Table 3, Table 4 and Table 5) and that the *ACAC* haplotype was 2.5 times more frequent in the group of THA patients (Table 7).

For our study, CA + L group results are important to note as they show not only the correlations for prostheses loosening but the tendency towards the total hip arthroplasty procedure overall. For *FokI*, *BsmI*, *TaqI* and *ApaI* (Table 2, Table 3, Table 4 and Table 5), *p*-values where <0.01, while haplotype analysis showed *ACAC* correlations (Table 6 and Table 7) in the CA + L and C groups (*p* < 0.001).

It is important to note that we found many studies linking bone health, BMD and osteoporosis; however, in most of them, the groups were significantly smaller and the statistical analyzes performed in the reviewed studies did not include ORs, calculated frequencies or full haplotype analyses. Comparisons based on percentages and numbers between groups without access to other data did not provide us with the right material for discussion and comparison with our data. Therefore, we summarize the available data in Table 8, where most of the important information was collected.

Vitamin 25(OH)D levels and deficiency have been well studied in many diseases and are the most commonly measured parameter for estimating vitamin D levels in the body due to its stability and long half-life [60,61]. Our results showed higher serum 25(OH)D levels in the C group (Figure 3) compared with the group of arthroscopy patients after THA without loosening of the prosthesis and patients after THA with loosened hip prostheses, which is consistent with the results of other researchers [16,62,63,64]. On the other hand, the mean value of the VDR serum concentrations is lower in the C group than in groups L and CA, although the values are higher in this group (Figure 4). In addition, the Spearman’s rank correlation Heat Map (Figure 5) showed a weak correlation between certain VDR and 25(OH)D levels, although the range of the C group suggests that 25(OH)D levels are higher on average in healthy patients. The results obtained suggest that VDR concentrations do not differ significantly between the tested and control groups. We could not find any other studies on VDR levels in similar study groups. Serum VDR levels may vary according to the physiological state of the body, depend on blood levels of 1,25(OH)2D and genetic variants [34,65,66] and may also show differential gene expression in different tissues. According to Verlinden et al. (2019), a role of osteoclastic *VDR* signaling in mice in the control of bone homeostasis has not been established, but in research by Nakamichi et al., 2017, the increase in bone mass was mediated by the suppression of bone resorption by VDR in osteoblasts [67,68].

## 4. Materials and Methods

### 4.1. Characteristics of Control and Patients Groups

A total of 374 Caucasian subjects were recruited between 2019 and 2024 at the The Voivodal Specialistic Hospital in Olsztyn, Poland. All required data were collected from patients as medical records and/or a completed questionnaire. All participants gave informed consent for the study, which was in accordance with ethical standards of the Declaration of Helsinki. The research was certified by the Bioethics Commissions (49/2019 and 139/2023).

The patients were divided into three groups: (1) 54 subjects from a group of patients with a hip transplant in whom the transplant was rejected (L—Loosening); (2) 76 subjects from a group of patients with a hip transplant in which the transplant is functioning correctly (CA—Control Arthroplasty); (3) 244 from a group of healthy volunteers (C—Control). The description of the group can be found in Table 9. ANOVA results showed there are significant differences in age among groups (F = 3.045, *p* = 0.0488). Post hoc Tukey (HSD) results indicated differences between groups, and they are presented in Table 9. There is a significant difference in age between CA and C. 

In groups L and CA, diagnosis was confirmed by clinical examination and radiographs, and both have a cementless prosthesis. Dominant symptoms of loosening were pain in lower limb on weight bearing, pain in the groin or thigh, new popping or clicking sounds, joint instability and dislocation or subluxation (partial dislocation) of the joint, which occurred 3 to 12 years after the initial prosthetic surgery. The diagnosis of loosening was based on insensitive radiographic criteria (periprosthetic radiolucent lines wider than 2 mm, prosthetic migration of more than 4 mm). Exclusion criteria included patients with a fistula, those who experienced a faulty surgical technique and those who did not fulfill the time criteria. Another exclusion criterion was patients’ BMI > 35. We did not include patients with osteoporosis and rheumatic diseases in the study group (these diseases have been shown to have an impact on the prosthesis survival). Therefore, we did not include patients with loosening who had fractures in this study.

### 4.2. Biological Material Collection

Approximately 5.0 mL of peripheral blood and 5.0 mL of serum were collected from a total of 374 participants. The biological material was immediately transported to the laboratory and used for analysis or stored at −80 °C. DNA was isolated using the Blood Mini DNA Isolation Kit and a purification procedure (A&A Biotechnology, Gdańsk, Poland) and diluted to a concentration of 20 ng/µL. The DNA was stored at −20 °C and awaited further analysis.

### 4.3. VDR Polymorphisms Genotyping

The mixture for amplification in a volume of 17.5 μL consisted of DreamTaq™ Green Master Mix (Thermo Scientific, Waltham, MA, USA), specific primers, the DNA matrix and nuclease-free water (Thermo Scientific, Waltham, MA, USA). The yield and specificity of the PCR products were evaluated by electrophoresis in 2.5% agarose gel (Promega, Fitchburg, WI, USA) and staining with GelGreen Nucleic Acid Gel Stain (Biotium, Fremont, CA, USA). The amplified fragments were digested with the restriction enzymes listed in Table 10 (Thermo Scientific, Waltham, MA, USA) according to the manufacturer’s instructions and visualized on a 2.5% agarose gel. DNA sequencing of randomly selected samples after amplification was performed to confirm correct genotyping by an external company. Table 10 shows primers, conditions, enzymes and amplicon characteristics. The methods were adapted from previous studies [69].

**Figure 6:**
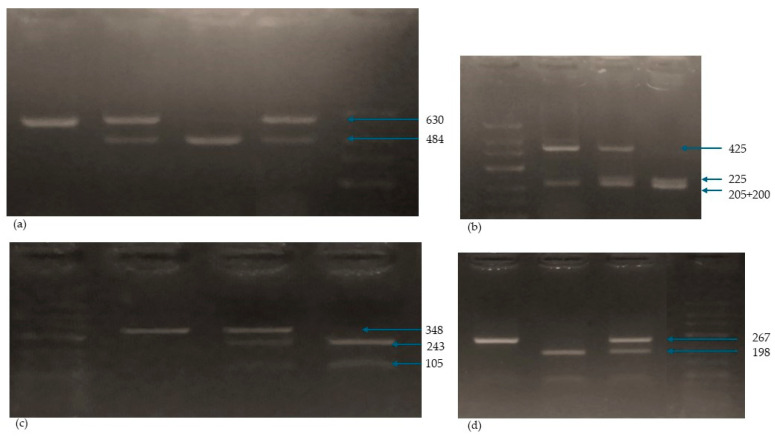
Electrophoregram of *Apa I* (**a**), *Taq I* (**b**), *Bsm I* (**c**) and *Fok I* (**d**) genotyping. *Apa*: Path 1: *AA* (630 bp), Path 2 and 4: *Aa* (630, 483 bp), Path 3: *aa* (484, 146 bp)–fragment of 146 bp not shown, Path 4: molecular marker. *Taq*: Path 1: molecular marker, Path 2: *TT* (425, 205 bp), Path 3: *Tt* (435, 225, 205, 200 bp), Path 4: *tt* (225, 205, 200 bp). Bsm: Path 1: molecular marker, Path 2: *BB* (348 bp), Path 3: *Bb* (348, 243, 105 bp), Path 4: *bb* (243, 105 bp). *Fok*: Path 1: *FF* (267 bp), Path 2: *ff* (198, 69 bp), Path 3: Ff (267, 198, 69 bp)–fragment of 69 bp not shown, Path 4: molecular marker.

Figure 6 shows the electropherograms of the *ApaI*, *TaqI*, *BsmI* and *FokI* genotypes.

### 4.4. VDR Serum Levels

Serum VDR levels were measured in two replicates using an ELISA kit (Biorbyt, UK). Statistical analysis was performed using GraphPad Prism 10.2.3 (GraphPad Software, Inc., Solana Beach, CA, USA). All kit components were stored at room temperature before use. The standard was reconstituted in 1.0 mL dilution buffer and shaken gently after 10 min to avoid foaming. Tubes containing 0.5 mL dilution buffer were used to dilute the standard solution, performing a double dilution series each time. The dilution points were 40 ng/mL, 20ng/mL, 10 ng/mL, 5ng/mL, 2.5 ng/mL, 0.625 ng/mL and blank 0 ng/mL. Reagents A and B were vortexed, centrifuged and diluted (1:100). The wash solution concentrate was diluted with distilled water (1:30). The samples were diluted (1:1) with PBS (pH = 7.1, 0.01 mol/L). All standard curve dilutions with blank and the analyzed samples were added to the plate with a volume of 100 μL and incubated at 37 °C for 2 h. The liquid was removed and 100 μL of the working solution of detection reagent A was added to each well. After a second 1 h incubation at 37 °C, the solution was aspirated and the plate was washed with 350 μL of wash solution (1 min). The liquid was removed and the plate was washed a total of 3 times. After the final third wash, the wash buffer was removed by decantating and blotted onto absorbent paper. The next step was the addition of 100 μL of Detection Reagent B to each well and the third incubation for 1 h at 37 °C. The aspiration and washing procedures were repeated 5 times with 350 μL of wash solution. After the last tapping against absorbent paper, 90 μL of substrate solution was added to each well and the plate was incubated at 37 °C for 15 min. Then, 50 μL of the stop solution was added to stop the reaction. The microplate was immediately illuminated with the Asys UVM-340 (Biochrome, Cambridge, UK) at 450 nm.

### 4.5. 25(OH)D Serum Levels

Serum levels of 25(OH)D were measured with an ELISA test in two repeats (Demeditec Diagnostics GmbH, Kiel, Germany). Statistical analysis was performed with GraphPad Prism. The standard curve of concentrations and two controls were prepared using the calibrators in the kit by reconstitution in 1 mL of distilled water. An amount of 25(OH)D conjugate concentrate was prepared at a dilution of 1:100. The HRP conjugate was prepared at a dilution of 1:200 approximately 2 h prior to use by mixing the following 3 reagents in the following order: conjugate buffer, concentrated buffer, vortex, concentrated HRP, vortex. The wash solution was diluted with water (1:200). An amount of 50 μL each of calibrator, control and sample were added to the appropriate wells together with 150 μL of the incubation buffer. The plate was incubated for 2 h at room temperature on a plate shaker (400 rpm). The liquid was aspirated, and the plate was washed 3 times with 350 μL of wash solution and aspirated each time. Then, 200 μL of working HRP conjugate solution was added into each well, and the plate was incubated for another 30 min at room temperature on a plate shaker (400 rpm). The liquid was aspirated and the plate was washed 3 times. The chromogenic solution was added at a volume of 100 μL. The plate was incubated for 15 min at room temperature on a plate shaker (400 rpm). After incubation, 100 μL of the stop solution was added into each well. The absorbance was measured at 450 nm with 650 nm reference immediately after addition of the stop solution.

### 4.6. Statistical Analysis

The relative association between allelic/genetic groups was assessed by calculating the odds ratios (ORs) and *p*-values in MedCalc “https://www.medcalc.org/calc/odds_ratio.php (accessed on 10 May 2024)”. ORs, as a measure of relative risk with 95% confidence intervals (95% CI), were estimated with logistic regression models and used to compare the allele frequencies in all of the studied groups. The LD and complete haplotype analyses (genotype distribution, Hardy–Weinberg equilibrium (HWE) and chi-square test) were performed in SHEsis online “http://shesisplus.bio-x.cn/SHEsis.html (accessed on 10 May 2024)”. Vitamin 25(OH)D and VDR concentration results were presented as means ± standard deviations (SD) (GraphPad Software, Inc., CA, USA). The means of the tested and control groups were compared using ANOVA and a post hoc Tukey (HSD) test was applied with *p* < 0.05 (GraphPad Software, Inc., CA, USA). A Spearman’s correlation test was performed using GraphPad Prism (GraphPad Software, Inc., CA, USA). 

## 5. Conclusions

In conclusion, this study indicates a possible genetic predisposition indicated by *VDR* polymorphisms associated with vitamin D deficiency in the etiology of prosthesis loosening. Our study found an association of *VDR* SNPs gene frequencies between the control group and the group of arthroscopy patients after THA and with THA but without prosthesis loosening. There was no association with VDR serum levels after surgery despite the differences in the occurrence of *VDR* genotypes, which may indicate that VDR serum levels may not be a good prognostic factor as they may vary according to the physiological state of the body.

## Figures and Tables

**Table 1:** Nomenclature of common polymorphisms in the VDR (Vitamin D Receptor) gene.

rs Number	SNP Name	Alleles
rs7975232	*ApaI* (Apa 1)	A (T), a (C)
rs1544410	*BsmI* (Bsm 1)	B (A), b (G)
rs2228570	*FokI* (Fok 1)	F (C), f (T)
rs731236	*TaqI* (Taq 1)	T (T), t (C)

**Table 2:** Genotype frequencies with logistic regression analysis of the dominant and recessive model of ApaI in VDR (Vitamin D Receptor) gene polymorphisms and χ^2^ in the studied groups and the associations with prosthesis loosening.

Genotypes	Control [*n* (%)] C	Control Arthroplasty [*n* (%)] CA	Loosening [*n* (%)] L
*AA*	53 (21.72)	19 (25.00)	12 (22.22)
*Aa*	128 (54.46)	44 (57.90)	30 (55.56)
*aa*	63 (25.82)	13 (17.10)	12 (22.22)
Genotype χ^2^ (4, 374) =2.54; *p* = 0.6375			
Frequency (F) of the alleles			
F*_A_*	0.48 (48)	0.54 (54)	0.50 (50)
F*_a_*	0.52 (52)	0.46 (46)	0.50 (50)
OR (95% CI; *p*-value)			
	C vs. CA + L	C vs. L	CA vs. L
dominant	0.68 (0.41–1.17; <0.0001)	0.82 (0.41–1.66; 0.0006)	1.39 (0.58–3.32; 0.0594)
recessive	0.88 (0.54–1.47; 0.0001)	0.97 (0.48–1.98; 0.0751)	1.17 (0.51–2.66; *p* = 0.71)
AA	-	-	-
vs. *Aa*	0.99 (0.58–1.68; 0.97)	1.04 (0.59–2.56; 0.03)	1.08 (0.46–2.55; 0.86)
vs *aa*	0.67 (0.36–1.29; 0.24)	0.84 (0.24–2.99; 0.0001)	1.46 (0.50–4.35; 0.49)

**Table 3:** Genotype frequencies with logistic regression analysis of the dominant and recessive model of TaqI in VDR (Vitamin D Receptor) gene polymorphisms and χ^2^ in the studied groups and the associations with prosthesis loosening.

Genotypes	Control [*n* (%)] C	Control Arthroplasty [*n* (%)] CA	Loosening [*n* (%)] L
*TT*	111 (45.49)	23 (30.26)	6 (11.11)
*Tt*	104 (42.62)	37 (48.69)	36 (66.67)
*tt*	29 (11.89)	16 (21.05)	12 (22.22)
Genotype χ^2^ (4, 374) *=* 25.924; *p* = 0.000033			
Frequency (F) of the alleles			
F*_T_*	0.67 (67)	0.55 (55)	0.56 (56)
F*_t_*	0.33 (33)	0.45 (45)	0.44 (44)
OR (95% CI; *p*-value)			
	C vs. CA + L	C vs. L	CA vs. L
dominant	2.04 (1.15–3.60; 0.0146)	1.13 (0.52–2.42; 0.75)	1.07 (1.46–2.49; 0.97)
recessive	2.91 (1.79–4.72; <0.0001)	6.68 (2.76–16.18; *p* < 0.0001)	3.47 (1.30–9.25; 0.01)
TT	-	-	-
vs. *Tt*	2.67 (1.62–4.46; 0.0001)	6.40 (2.59–15.83; 0.0001)	1.72 (0.97–3.08; 0.07)
vs. *tt*	3.70 (1.91–7.17; 0.0001)	7.66 (2.65–22.14; 0.0002)	2.88 (0.89–9.36; 0.08)

**Table 4:** Genotype frequencies with logistic regression analysis of the dominant and recessive model of BsmI in VDR (Vitamin D Receptor) gene polymorphisms and χ^2^ in the studied groups and the associations with prosthesis loosening.

Genotypes	Control [*n* (%)] C	Control Arthroplasty [*n* (%)] CA	Loosening [*n* (%)] L
*BB*	33 (13.52)	15 (19.74)	13 (24.07)
*Bb*	145 (59.43)	48 (63.15)	35 (64.82)
*bb*	66 (27.05)	13 (17.11)	6 (11.11)
Genotype χ^2^ (4, 374) = 10.258; *p =* 0.0363			
Frequency (F) of the alleles			
F*_B_*	0.56 (56)	0.51 (51)	0.56 (56)
F*_b_*	0.44 (44)	0.49 (49)	0.44 (44)
OR (95% CI; *p*-value)			
	C vs. CA + L	C vs. L	CA vs. L
dominant	0.46 (0.26–0.81; 0.0071)	0.34 (0.14–0.82; 0.02)	0.61 (0.32–1.71; 0.34)
recessive	0.57 (0.33–0.99; 0.0475)	0.49 (0.24–1.01; 0.06)	0.78 (0.33–1.80; 0.55)
BB	-	-	-
vs. *Bb*	0.67 (0.38–1.20; 0.18)	0.62 (0.29–1.29; 0.20)	0.84 (0.36–1.99; 0.69)
vs. *bb*	0.34 (0.17–0.70; 0.0031)	0.23 (0.08–0.66; 0.0064)	0.53 (0.16–1.80; 0.31)

**Table 5:** Genotype frequencies with logistic regression analysis of the dominant and recessive model of FokI in VDR (Vitamin D Receptor) gene polymorphisms and χ^2^ in the studied groups and the associations with prosthesis loosening.

Genotypes	Control [*n* (%)] C	Control Arthroplasty [*n* (%)] CA	Loosening [*n* (%)] L
*FF*	17 (6.97)	16 (21.05)	12 (22.22)
*Ff*	108 (44.26)	40 (52.63)	30 (55.56)
*ff*	119 (48.77)	20 (26.32)	12 (22.22)
		Genotype χ^2^ (4, 374) = 29.084; *p* < 0.00001	
Frequency (F) of the alleles			
F*_F_*	0.29 (29)	0.47 (48)	0.50 (50)
F*_f_*	0.71 (71)	0.53 (52)	0.50 (50)
OR (95% CI; *p*-value)			
	C vs. CA + L	C vs. L	CA vs. L
dominant	0.34 (0.21–0.55; <0.0001)	0.30 (0.15–0.60; 0.0006)	0.80 (0.35–1.81; 1.0)
recessive	0.27 (0.15–0.52; 0.0001)	0.26 (0.21–1.08; 0.08)	0.93 (0.40–2.18; 1.0)
FF	-	-	-
vs. *Ff*	0.39 (0.20–0.77; 0.0066)	0.39 (0.17–0.91; 0.03)	1.00 (0.41–2.42; 1.0)
vs. *ff*	0.16 (0.08–0.33; <0.0001)	0.14 (0.06–0.37; 0.0001)	0.80 (0.28–2.25; 0.67)

**Table 6:** Haplotype analysis of the loci *ApaI*, *TaqI*, *BsmI* and *FokI* SNPs in the *VDR* (*Vitamin D Receptor*) gene in the groups L vs. CA + C.

Haplotype	Case (Freq)	Control (Freq)	χ^2^	Fisher’s *p*	Pearson’s *p*	OR [95% CI]
*ACAT*	11 (0.101)	80 (0.125)	0.463	0.632	0.496	0.79 [0.41–1.55]
*ACAC*	28 (0.259)	83 (0.129)	12.276	0.001	0.0004	2.35 [1.44–3.83]
*CTGT*	23 (0.212)	178 (0.278)	1.996	0.196	0.157	0.70 [0.43–1.15]
*ATGT*	6 (0.055)	79 (0.123)	4.227	0.047	0.039	0.42 [0.18–0.98]
*CTAT*	7 (0.064)	26 (0.040)	1.282	0.305	0.257	1.64 [0.69–2.87]
*ATAT*	1 (0.009)	30 (0.046)	3.291	0.07	0.069	0.19 [0.03–1.41]
*ATAC*	2 (0.018)	23 (0.035)	0.867	0.561	0.351	0.51 [0.12–2.18]
*CCAC*	5 (0.046)	18 (0.028)	1.023	0.359	0.311	1.68 [0.61–4.62]
*CTGC*	5 (0.046)	50 (0.078)	1.374	0.319	0.241	0.57 [0.22–1.47]

Haplotypes with frequency < 0.03 were ignored. Global result: total control = 320, total case = 54. Global χ^2^ is 24.452, Fisher’s *p* is NA, Pearson’s *p* is 0.001.

**Table 7:** Haplotype analysis of the loci *ApaI*, *TaqI*, *BsmI* and *FokI* SNPs in the *VDR* (*Vitamin D Receptor*) gene in groups L vs. C.

Haplotype	Case (Freq)	Control (Freq)	χ^2^	Fisher’s *p*	Pearson’s *p*	OR [95% CI]
*ACAT*	11 (0.101)	64 (0.131)	0.689	0.521	0.406	0.75 [0.38–1.48]
*ACAC*	28 (0.259)	60 (0.122)	13.055	0.0008	0.0003	2.50 [1.50–4.15]
*CTGT*	21 (0.194)	151 (0.309)	5.694	0.018	0.017	0.54 [0.32–0.9]
*ATGT*	6 (0.055)	65 (0.133)	5.079	0.021	0.024	0.38 [0.16–0.91]
*CTAT*	7 (0.064)	21 (0.043)	0.937	0.319	0.333	1.54 [0.64–3.72]
*CTGC*	7 (0.064)	41 (0.084)	0.440	0.695	0.506	0.76 [0.33–1.73]
*ATAT*	1 (0.009)	24 (0.049)	3.506	0.064	0.061	0.18 [0.02–1.35]

Haplotypes with frequency <0.03 were ignored. Total control = 244, total case = 54. Global χ^2^ is 24.448, Fisher’s *p* is NA, Pearson’s *p* is 0.0004.

**Table 8:** Summary of studies investigating *VDR* role in bone diseases.

Results	Study Cohort	Case	References
*FokI*—*Ff* genotype strongly associated with femoral hip BMD compared to the control group, which had the lowest hip BMD; *TaqI*—*TT* genotype associated with the lowest hip BMD compared to the control; *tt* genotype—highest hip BMD *ApaI*—*Aa* correlated with the lowest mean of BMD; *aa* genotype correlated with highest hip BMD; *BsmI*—*bb* genotype correlated with the lowest hip BMD, while the *BB* genotype had the highest hip BMD.	*N* = 65 of osteoporosis patients; *N* = 30 controls	BMD	[40]
*ApaI*—No significant difference was found between the different genotypes with regard to the occurrence of osteoporosis with osteoporotic fractures.	Total of 378 patients *N* = 235 BMD cases; *N* = 65 decreasing BMD patients	BMD	[50]
The genotypes of *BsmI*, *TaqI*, and *FokI* were not significantly associated with BMD; *ApaI*—*AA* genotype had a higher lumbar spine BMD than the individuals with the *aa VDR* genotype.	*N* = 83	BMD	[51]
*ApaI*—BMD was lower in patients with genotype aa compared to genotype *AA*.	*N* = 136 woman (75—osteoporosis, 37—osteopenia and 24—normal BMD)	BMD	[46]
*Bsm/Apa/Taq BB/AA/tt* (*AATTCC)* haplotype was a risk factor for OP (OR: 5.66), while *BbaaTT (AGCCTT)* had a protective effect (OR = 0.10).	*N* = 147 postmenopausic women; (71 OP and 76 controls)	BMD	[52]
*VDR* polymorphisms are not associated with the risk of hip fractures.	*N* = 126 (67 with fractures, 59 healthy)	BMD	[53]
*FokI* is associated with osteoporosis (OR_ff+Ff vs. FF_ = 1.19); *TaqI* is associated with osteoporosis (OR_TT+Tt vs. tt_ = 1.35).	*N* = 6880 cases and *n* = 8049 controls (meta-analysis)	BMD	[45]
*TaqI*—*CC* (*tt*) genotype associated with increased risk of hip fracture (OR = 2.6; 95%CI = 1.2–5.3) and BMD.	*N* = 677 postmenopausal women, *n* = 69 hip fracture status	BMD	[47]
*TaqI*—low BMD has been associated with the *tt* (or *AA* or *BB*) genotype.	*N* = 43 osteoporotic postmenopausal women *N* = 139 healthy women	BMD	[54]
No association between the *VDR* genotype and osteoporosis.	*N* = 118 premenopausal women	BMD	[44]
*BsmI*—(*GG*) *bb* genotype had a femoral neck bone density of 0.79 standard deviations lower than individuals with *BB* genotype.	*N* = 44 osteoporosis; *N* = 44 controls	BMD	[55]
*ApaI*, *BsmI*, *TaqI*—no correlation between the *VDR* genotypes and BMD.	*N* = 84 osteoporosis woman *N* = 807 controls	BMD	[56]
*ApaI* and *FokI*—no association between genotypes and BMD *BsmI*—*BB + Bb* genotypes more frequent in patients with osteoporotic fractures; *TaqI*—*TT*-genotyped patients had a higher BMD compared to *Tt* or *tt*.	*N* = 192 osteoporosis patients, = 207 controls	BMD	[57]
*FokI*—(*TT*) *ff* genotype had a significantly lower BMD at the hip than those with the *Ff* genotype; the difference between the two homozygous genotypes (*FF* vs. *ff*) was not significant at any point; *ApaI*, *BsmI* and *TaqI* polymorphisms were not associated with BMD.	N = 114 postmenopausal women including *n* = 33 healthy controls and *n*= 65 osteoporotic	BMD	[58]
*TaqI* with *T* allele was significantly associated with BMD, while the *Apa aa* variant, the *Bsm bb* variant and the *TT Taq* variant occurred most frequently in groups with higher fracture risk.	*N* = 187 osteoporotic patients, *n* = 19 controls	BMD	[48]
*Bsm/Apa/Taq: bbAATT* and *bbTtAa* were more frequent in the osteoporosis group compared to the healthy control group.	*n* = 200 with osteoporosis, *n* = 146 healthy controls	BMD	[49]
*FokI*—(*F*) *C* allele nearly associated with the development of osteoporosis compared to control (OR = 1.783, 95%CI = 0.98–3.25, *p* = 0.058).	*N*—120 postmernopausal women (*n* = 88 non osteoporotic, 144—osteopenic, *n* = 88 osteoporotic)	BMD	[59]

**Table 9:** Patients characteristics.

Characteristics	L (*n* = 54)	CA (*n* = 76)	C (*n* = 244)	*p*-Value (L vs. CA/L vs. C/CA vs. C)
Age: years mean (SD)	52.7 (9.0)	54.0 (10.8)	51 (9.5)	0.74/0.46/0.05 *
Gender: *n* (%)				
Female	22 (41)	19 (25)	120 (49)	
Male	32 (59)	57 (75)	124 (51)	
χ^2^ (2, 374) = 13.9573				0.0009

* post hoc test: Tukey (HSD).

**Table 10:** PCR primers, conditions, enzymes used and amplicon characteristics.

SNP	Primer Sequence for PCR	Enzyme	PCR Conditions	PCR-RFLP Amplicon
Fok-I rs2228570	Fok1R: 5-ATGGAAACACCTTGCTTCTTCTCCCTC-3	FokI	Initial denaturation: 94 °C/3 min denaturation: 94 °C/30 s. annealing: 61 °C/30 s. synthesis: 72 °C/30 s. number of cycles: 35	ff: 198, 69 FF: 267 Ff: 267, 198, 69 PCR product: 267
	Fok11F: 5-AGCTGGCCCTGGCACTGACTCtGGCTCT-3			
Bsm-I rs1544410	Bsm1F: 5-CGGGGAGTATGAAGGACAAA-3	BsmI	Initial denaturation: 94 °C/3 min denaturation: 94 °C/30 s. annealing: 64 °C/30 s. synthesis: 72 °C/30 s. number of cycles: 35	bb: 243, 105 BB: 348 Bb: 348, 243, 105 PCR product: 348
	Bsm1R: 5-CCATCTCTCAGGCTCCAAAG-3			
Taq-I rs731236	Taq1F: 5-GGATCCTAAATGCACGGAGA-3 Taq1R: 5-AGGAAAGGGGTTAGGTTGGA-3	TaqI	Initial denaturation: 94 °C/3 min denaturation: 94 °C/30 s. annealing: 62 °C/30 s. synthesis: 72 °C/30 s. number of cycles: 35	tt: 225, 200, 205 TT: 425, 205 Tt: 425, 225, 200, 205 PCR product: 630
Apa-I rs7975232		ApaI		aa: 484, 146 AA: 630 Aa: 630, 484, 146 PCR product: 630

## Data Availability

Detailed information is available upon request from the corresponding author.
